# Dental Pulp Stem Cells for Bone Tissue Engineering: A Literature Review

**DOI:** 10.1155/2023/7357179

**Published:** 2023-10-11

**Authors:** Xiaolei Bai, Ruijue Cao, Danni Wu, Huicong Zhang, Fan Yang, Linhong Wang

**Affiliations:** ^1^Department of Stomatology, Zhejiang Provincial People's Hospital, Affiliated People's Hospital, Hangzhou Medical College, Hangzhou 310018, Zhejiang, China; ^2^Center for Plastic & Reconstructive Surgery, Department of Stomatology, Zhejiang Provincial People's Hospital, Affiliated People's Hospital, Hangzhou Medical College, Hangzhou 310018, Zhejiang, China

## Abstract

Bone tissue engineering (BTE) is a promising approach for repairing and regenerating damaged bone tissue, using stem cells and scaffold structures. Among various stem cell sources, dental pulp stem cells (DPSCs) have emerged as a potential candidate due to their multipotential capabilities, ability to undergo osteogenic differentiation, low immunogenicity, and ease of isolation. This article reviews the biological characteristics of DPSCs, their potential for BTE, and the underlying transcription factors and signaling pathways involved in osteogenic differentiation; it also highlights the application of DPSCs in inducing scaffold tissues for bone regeneration and summarizes animal and clinical studies conducted in this field. This review demonstrates the potential of DPSC-based BTE for effective bone repair and regeneration, with implications for clinical translation.

## 1. Introduction

**Figure 1:**
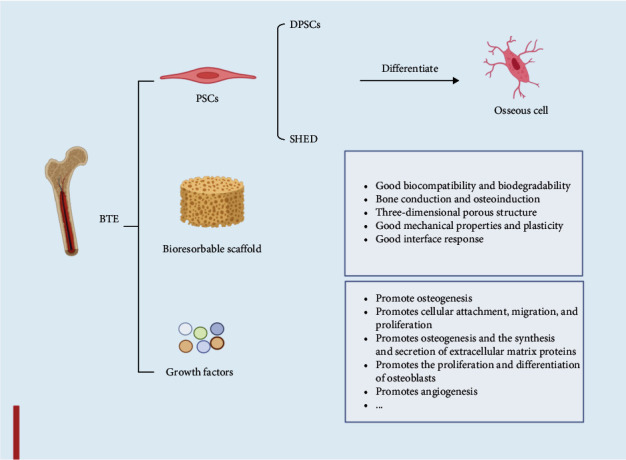
Three key elements of bone tissue engineering: stem cells capable of differentiating into osteoblasts, regulated by inducible growth factors, and bioresorbable scaffolding matrices to support cellular attachment, migration, and proliferation; promote osteogenesis; promotes osteogenesis and the synthesis and secretion of extracellular matrix proteins, such as collagen; promotes the proliferation and differentiation of osteoblasts; and promotes angiogenesis.

Bone tissue engineering (BTE) aims to leverage bone stem cells to regenerate, repair, and remodel bone tissue in response to mechanical stimulation and injury [1, 2]. Various therapeutic approaches have been employed for tissue-engineered repair of bone defects, which aim to replicate the natural process of bone repair by delivering a source of stem cells capable of differentiating into osteoblasts, as well as inductive growth and differentiation factors, and bioresorbable scaffolding matrices that can support cellular attachment, migration, and proliferation [3, 4] (Figure 1). Stem cells exhibit varying osteogenic differentiation potential based on their origin from distinct tissues [5–7] and include induced pluripotent stem cells, embryonic stem cells, and somatic stem cells. The application of BTE for bone defect repair has made significant strides in recent years. However, many challenges must be addressed before it can be widely utilized in clinical practice. The most used stem cell source for BTE is bone marrow stem cells (BMSCs) that belong to the mesenchymal stem cells (MSCs) category of somatic stem cells, which exhibit robust osteogenic differentiation capabilities. Nevertheless, there are several drawbacks of using BMSCs, such as the need for invasive donor procedures that can result in considerable trauma [8, 9], a limited yield of cells [10, 11], and reduced stem cell differentiation potential due to donor age [12, 13], which need further discussion [14].

Dental pulp stem cells (DPSCs) are a promising and versatile source for bone regeneration. DPSCs include DPSCs isolated from permanent teeth and stem cells from human exfoliated deciduous teeth (SHED), both of which possess the capacity for multilineage differentiation and have a high proliferation rate, maintaining their multipotency even when expanded in vitro. DPSCs possess a high osteogenic potential and can differentiate into osteoblasts, promote angiogenesis, and modulate immune function, all important for promoting new bone formation. DPSCs also have the unique ability to form functional dentin–pulp complexes, making them a promising source for complex tissue regeneration. Compared with BMSCs [15], DPSCs have the advantages of facile extraction from dental pulp tissue, convenient preservation, low immune prototype, and minimal ethical controversy. DPSCs are derived from the neural crest and exhibit unique neurogenicity [16]. The neural crest is a collection of cells arising from the embryonic ectoderm [17, 18]. At the end of the third week of embryonic development, the notochord induces the differentiation of some ectodermal cells to form a neural plate. Cells at the edge of the neural plate begin to form neural folds that, as they grow and bulge, converge on each other to produce neural grooves. At the edges of the neural folds, some cells begin to proliferate and form neural crests. The neural crest initially lies between the epidermis and the neural tube and then begins to migrate in different directions, becoming the building blocks for the formation of tissues and organs such as the teeth, the nervous system, and the facial skeleton. The formation and movement of the neural crest are controlled and regulated through complex signaling pathways and molecular mechanisms. Its formation can be divided into two steps: regulation by signaling molecules that control gene expression [19] and movement and differentiation to form different tissues and organs [18]. Cells derived from the neural crest participate in tooth development and reside in the pulpal connective tissue until adulthood and can also maintain their stemness [20].

In terms of neurogenesis, DPSCs could produce neuron-like cells [21, 22] and neurotrophic factors [23], which can stimulate nerve regeneration and growth [24]. They also help promote the regeneration of Schwann cells [25], a significant nerve cell type, which contribute to the functional repair and regeneration of the nervous system. Nerve regeneration plays an important role in the process of bone tissue regeneration [26–28]: nerve growth can affect the migration, proliferation, and differentiation of bone cells and promote the regeneration and repair of bone tissue; bone injury is often accompanied by nerve injury. Restoring nerve function can avoid bone tissue dysfunction and delayed repair. In addition, endogenous neuromodulators, such as nerve growth factor [21] and neurotrophic factor [23], can regulate bone cells and bone regeneration, promote bone cell proliferation, differentiation, and growth, and play an important role in angiogenesis [29, 30] and bone tissue formation. There is a complex relationship between the nervous system and bone tissue. The nerve plays a key role in the development and growth of bone, and bone tissue can also affect nerve signal transduction [31] and nerve cell development through cell secretion and mechanical force. DPSCs are a potential source of stem cells that can differentiate into osteocytes and neural-like cells to achieve simultaneous repair of bone and nerve [32]. Certain nerve growth factors and specific proteins produced during osteogenesis have been found to further enhance osteogenic differentiation through overlapping signaling pathways [33]. In comparison to MSCs derived from dental pulp, dental follicle, and dental papilla of the same tooth (Table 1), DPSCs are more neurogenic [34]. Gronthos et al. [35] first reported the isolation of DPSCs from the dental pulp tissue of human third molars. Subsequent research by Shi et al. [36] demonstrated that DPSCs exhibit higher CFU-F and proliferation rates, as well as similar gene expression profiles of mineralization-related genes. In vitro, expansion of DPSCs transplantation leads to the formation of a dentin–pulp-like structure, while in vivo transplantation of BMSCs forms heterotopic bone [37]. Shortly, thereafter, Miura et al.'s study [15] revealed that SHED, which are extracted from exfoliated deciduous dental pulp, have a greater capacity to induce osteogenesis than DPSCs. Subsequent studies have demonstrated that both DPSCs and SHED possess strong proliferation, self-renewal ability, and multidirectional differentiation potential. These attributes confer unique advantages in osteogenesis.

Therefore, an in-depth understanding of the osteogenic potential of DPSCs holds immense significance for researchers to comprehend the entire spectrum of bone regeneration and reconstruction. Accordingly, this review provides a comprehensive analysis of the biological characteristics of DPSCs, including multipotent differentiation ability, proliferation and renewal ability, and immune regulation ability, as well as the mechanism and application of regulating DPSCs cell fate in osteogenesis.

## 2. Biological Characteristics

### 2.1. Stem Cell Sources and Isolation Methods (Table 2)

DPSCs, which are derived from teeth that have been clinically extracted and discarded, can be effectively isolated using either the tissue block method or the enzyme digestion method [38]. The DPSCs obtained through the enzyme digestion method exhibit superior clone formation rates and proliferation abilities when compared to those obtained through the tissue block method. Because SHED has many similar biological characteristics to DPSCs, the SHED can be obtained by the same method. Following separation from dental pulp tissue, DPSCs can be screened for high proliferation potential, surface markers, and nuclear staining through high-throughput fluorescence. At present, cryopreservation is a commonly used method for storing DPSCs, but the disadvantages are time-consuming [39]. However, the new cryopreservation method (NCM) allows for the use of frozen pulp tissue for the extraction of DPSCs after thawing without impacting their value-added ability and significantly reducing costs compared to cryopreservation [40]. DPSCs can be stored in a serum-free cryopreserved suspension in a refrigerator at −80°C for up to 1 year [41]. After resuscitation, they can still differentiate into multiple directions and maintain their original cell morphology through the 10th generation of cell culture [22]. Due to their multilineage differentiation potential, DPSCs and SHED are regarded as candidates for bone regeneration.

### 2.2. Multipotential Differentiation Potential

**Figure 2:**
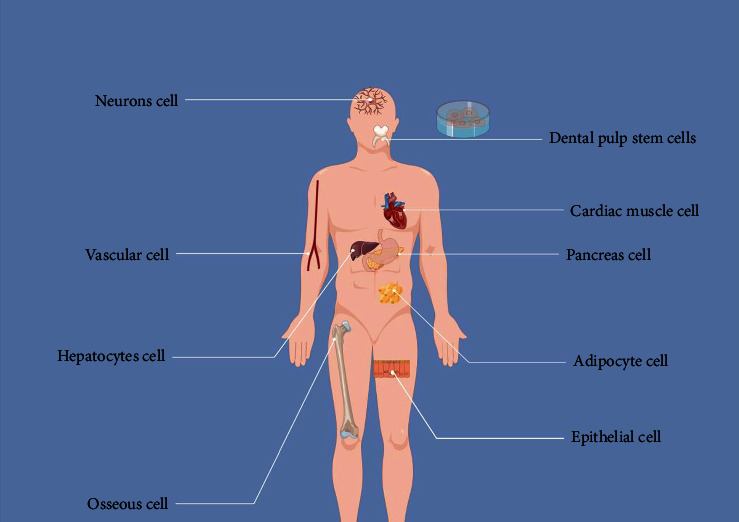
Differentiation potential of dental pulp stem cells.

DPSCs possess the capacity for multilineage differentiation. Recent research has demonstrated that DPSCs can differentiate into various cell types, including odontoblasts, adipocytes, osteoblasts, neuronal cells, chondrocytes, muscle cells, hepatocytes, and pancreatic cells, in response to specific induction cues [42–45] (Figure 2). However, the differentiation potential of DPSCs is governed by gene expression profiles [46]. For example, DPSCs isolated from permanent teeth are more prone to neuronal lineage differentiation, whereas SHED exhibits superior differentiation potential toward bone and adipose tissue [47]. Thus, for clinical applications, choosing DPSCs with favorable gene expression patterns for specific lineage differentiation holds great promise for the development of diversified and effective therapies.

### 2.3. Proliferation and Self-Renewal Ability

The maintenance of tissue and organ homeostasis and regeneration relies on a complex interplay of cellular processes, including proliferation, migration, adhesion, and differentiation [48, 49]. Notably, DPSCs have been shown to exhibit a significantly higher clonal proliferation rate than BMSCs, with this heightened capacity remaining robust through passages [16, 35]. Furthermore, DPSCs share common features with MSCs, including a potent and stable self-metabolic ability, as well as a remarkable degree of plasticity, that is subject to the regulatory control of other factors [49]. Self-renewal ability is manifested in proliferation in vivo to maintain its number, and clonal growth can be cultured in vitro. It can be evaluated by colony-forming unit assay. SHED was found to be more efficient than DPSC in colony formation [50]. Of course, self-renewal is a double-edged sword [51]. On the bright side, it can ensure that the stem cell population is not depleted over time, thus providing an inexhaustible source of cell replacement in vivo and treatment. On the dark side, self-renewal-driven machines may be hijacked by transformed cells to achieve the replication of immortality-triggering tumors.

### 2.4. Immunoregulation

DPSCs interact with various components of the innate immune system, adaptive immune system, and complement system [2, 52–54]. Specifically, SHED has been found to inhibit the proliferation of Th17 cells in vitro [52] and reverse immune disorders in conditions such as systemic lupus erythematosus by increasing the proportion of regulatory T cells in the body by acting on Th17 cells [55]. Similarly, DPSCs have been shown to induce apoptosis of activated T cells and the related tissue injury [56, 57], as well as inhibit B-cell proliferation in mixed lymphocyte reactions. The low expression of MHC II indicates their low antigen reactivity [40]. Furthermore, DPSCs treated with lipid phosphate walls have been found to express nearly all the factors required to activate the complement system [58] and can further proliferate and activate DPSCs by expressing factors such as C3a and C5a [59]. The negative immune regulation, low immunogenicity, and immune tolerance of DPSCs make them a promising candidate for tissue engineering and bone regeneration. Studies have shown that DPSCs can interact with macrophages, a type of immune cell that plays a crucial role in the body's defense against infections [43, 60]. Several ways in which DPSCs interact with macrophages include: secreting chemokines and cytokines to attract macrophages to injury or inflammation sites, regulating the function of macrophages [61], and promoting their polarization to proinflammatory or anti-inflammatory directions [60]. DPSCs can also promote the polarization of macrophages to M2 phenotype [58], which can alleviate neurological damage and reduce the neuroinflammatory response caused by oxidative stress and abnormal homeostasis after peripheral nerve injury to a certain extent [44]. In summary, the interaction between DPSCs and macrophages with immunomodulatory properties can facilitate tissue repair and regeneration processes such as bone regeneration and wound healing.

However, most of the experiments regarding the immune characteristics of DPSCs are conducted in vitro or on animal models, and the regulation mechanism of complex humoral factors in vivo remains unclear. Further research is necessary to better understand the immune properties of DPSCs within the human body.

## 3. Osteogenic Differentiation Ability

The osteogenic differentiation potential of DPSCs has been widely confirmed in the literature. A cDNA microarray analysis has demonstrated that DPSCs can participate in the formation of craniofacial structures, including craniofacial bones and cartilage [36]. In vitro, DPSCs express osteogenic protein markers, including bone sialoprotein (BSP), alkaline phosphatase (ALP), and dentin sialoprotein (DSP), which enable them to differentiate into osteoblasts [62]. Moreover, DPSCs exhibit high expression of specific markers of MSCs, such as CD13, CD29, CD44, CD59, CD73, CD90, CD105, CD106, CD146, CD166, CD271, STRO-1, and STRO-3 [63–68]. DPSCs also express various osteogenic-related proteins, including ALP, type I collagen, bone morphogenetic protein 2 (BMP2), bone morphogenetic protein 4 (BMP4), osteonectin, osteopontin (OPN), and osteocalcin (OCN), and fibroblast-related proteins, including type III collagen and fibroblast growth factor (FGF)-2 [50, 56]. The absence of BSP and dentin sialo phosphoprotein (DSPP) was detected in DPSCs culture, which could indirectly reflect the absence of differentiation [14, 22]. Under the influence of an osteogenic medium, DPSCs can induce the formation of mineralized nodules [69]. ALP is an early indicator of osteoblast differentiation and participates in the formation, metabolism, and regeneration of calcified tissues such as bone. The higher the ALP activity, the more pronounced the osteogenic differentiation of cells [50, 70, 71]. The essence of the osteogenic differentiation of DPSCs is their ability to differentiate into osteoblasts with mineralized properties. The mechanism of differentiation regulation is similar to that of odontogenic differentiation, and the process is precisely regulated by genes and growth factors [72].

### 3.1. Growth Factors

The differentiation process of DPSCs plays a crucial role in promoting bone regeneration, which is facilitated by a plethora of growth factors. In particular, the osteogenic differentiation of DPSCs is closely linked to the activity of several growth factors (Table 3), including the subfamily of transforming growth factor-*β* (TGF-*β*), the subfamily of bone morphogenic proteins (BMPs), as well as other factors such as basic fibroblast growth factor (bFGF), nerve growth factor (NGF), and platelet-derived growth factor (PDGF).

TGF-*β* is a multifunctional protein with regulatory properties that plays a critical role in promoting osteoblast proliferation, inhibiting apoptosis, and regulating osteoclast activity at appropriate concentrations. Notably, a synergistic effect exists between TGF-*β*1 and BMP2, as demonstrated in mouse embryonic osteoblasts, where the expression of key osteogenic markers such as ALP, collagen I, and OCN was significantly upregulated in a dose-dependent manner after treatment with both factors [73]. Furthermore, the concentration of TGF-*β*2 at 1 ng/mL has been shown to promote the strongest proliferation, osteogenic differentiation, and mineralization of BMSCs [74]. A dose-dependent increase in the expression of OCN and COL I was observed in DPSCs treated with TGF-*β* [75]. In addition, TGF-*β* has been found to inhibit the expression of TNF-*α* and upregulate the expression of osteoprotegerin (OPG), thereby inhibiting the activity of osteoclasts and indirectly promoting osteogenesis [76]. Currently, there are four TGF-*β* subtypes (TGF-*β*1, TGF-*β*2, TGF-*β*3, TGF-*β*1*β*2), each with distinct gene loci and biological effects. Therefore, investigating the association between each subtype and other growth proteins in bone formation is a valuable area for further research.

With regard to the BMP subfamily, BMP2 [77], BMP4 [78], BMP7 [79], and BMP9 [80] have been proven to have osteogenic effects in the literature. Notably, the concentration of BMP2 [59] at 10 ng/mL was found to have the strongest induction effect in a study investigating the combined effect of vascular endothelial growth factor (VEGF) and BMP2 on the osteogenic differentiation of DPSCs [59]. It should be noted that VEGF also exhibits good osteogenic induction activity, and the synergistic effect of VEGF with BMP2 occurs only in the early stage of osteogenic differentiation (<7 days), after which it inhibits the activity of BMP2. Overexpressed BMP2 and BMP7 in human dental germ stem cells could promote osteogenic differentiation and odontogenic differentiation and both of them could promote each other [79]. Meanwhile, BMP2 is considered the most potent factor in the BMP family and plays a pivotal role in bone formation, as it promotes the osteogenesis of DPSCs dependent on the core binding factor A2T2 [81]. Accordingly, when the factor is silenced, the interaction of autosomal histone methyltransferase 1 promotes the expression of EHMT1 and promotes the methylation of H3K9me2, thereby inhibiting the expression of Runx2 promoter and promoting the osteogenesis of DPSCs [63].

bFGF is capable of inducing mitosis of most mesenchymal and neuroectodermal cells, stabilizing the phenotype of cultured cells, promoting cell proliferation and migration, and exhibiting a wide range of biological effects [31, 82]. When DPSCs were cultured in the presence of bFGF, the number of mitotic S-phase cells was significantly increased, and the expression of stem cell marker STRO-1 was higher than that in the non-bFGF group [65]. The effect of bFGF on the osteogenic differentiation of DPSCs is time-dependent [83, 84], that is, induction of DPSCs with bFGF for 1 week can promote their osteogenic differentiation, whereas induction for 2 weeks inhibits osteogenesis both in vivo and in vitro. Additionally, bFGF has a dose-dependent inhibition mechanism [85], whereby 1–5 *μ*g/L bFGF can promote the ALP expression of DPSCs, while 10–500 *μ*g/L bFGF can inhibit both ALP expression and mineralization ability in vitro.

NGF is a cytokine involved in nerve development and regeneration [16, 86]. It also plays an important role in promoting bone metabolism and osteogenic differentiation. When combined with DPSCs, NGF can promote the proliferation of bone cells [87].

Additionally, PDGF is a polypeptide growth factor found in platelets and plays a crucial role in maintaining the stability of neovascularization. PDGF-BB promotes the chemotaxis of MSCs, which is closely related to the proliferation and differentiation of DPSCs [88, 89]. Moreover, the combination of PDGF with other growth factors can improve the induction effect [72].

### 3.2. Osteogenesis Mechanism

**Figure 3:**
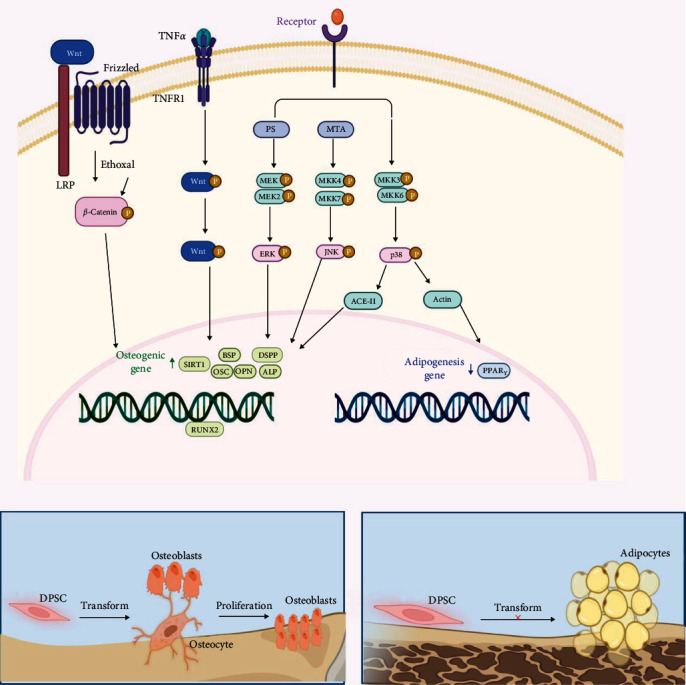
Osteogenic differentiation-related signal pathway diagram. The activation of the Wnt signaling pathway leads to the accumulation of *β*-catenin. TNF-*α* can enhance the expression of the Wnt signaling pathway agonist SIRT1, thereby activating this pathway and promoting osteogenic differentiation of DPSCs. ERK1/2 pathway regulates phosphatidylserine (PS) and upregulates the expression of related osteogenic genes. P38 MAPK is involved in the regulation of ACE-II-induced proliferation, and actin inhibitor-mediated osteogenesis inhibits adipogenic differentiation. c-Jun *N*-terminal kinase signaling pathway regulates methionine adenosyltransferase and promotes the expression of osteogenic genes.

The differentiation of DPSCs into osteoblasts is a complex process that involves the regulation of multiple signaling pathways, including the Wnt/*β*-catenin signaling pathway and the mitogen-activated protein kinase (MAPK) pathway (Figure 3).

The Wnt/*β*-catenin signaling pathway plays an important role in cell proliferation and differentiation during embryogenesis, postnatal development, and tissue homeostasis [90]. It is also important for maintaining stem cell stability [77] and expansion [78]. The pathway is also involved in regulating the osteogenesis process [91]. Activation of the Wnt signaling pathway leads to the accumulation of *β*-catenin, which regulates the osteogenic differentiation of SHED [92]. Moreover, TNF-*α* has been shown to enhance the expression of the Wnt signaling pathway agonist SIRT1, which activates the Wnt/*β*-catenin signaling pathway and promotes osteogenic differentiation of DPSCs [93]. The biological role of the Wnt signaling pathway in regulating the differentiation of DPSCs into osteoblasts is complex, often requiring coordination with other pathways. For instance, the MAPK signal transduction pathway is frequently involved, as discussed further below.

It has been well documented that the MAPK family includes extracellular signal-regulated kinase (ERK)1/2, p38 MAPK, and c-Jun *N*-terminal kinase (JNK), which are particularly closely related to stem cell research. First, the activation of the ERK1/2 signaling pathway promotes the proliferation and osteogenic differentiation of human MSCs [94]. ERK1/2 pathway regulates phosphatidylserine to promote the formation of mineralized calcium nodules in stem cells, upregulate ALP activity and the expression of related osteogenic genes, and enhance the osteogenic differentiation potential of stem cells [95]. Then, p38 MAPK is involved in the regulation of angiotensin II-induced proliferation [96] and differentiation of pluripotent stem cells into mesodermal progenitor cells, and also plays a role in actin inhibitor-mediated osteogenic [97] and adipogenic differentiation of MSCs. The insulin-like growth factor-1 receptor and p38 MAPK maintain the quiescent state of DPSCs and promote their proliferation, differentiation, and self-renewal through opposite transduction signaling pathways [98]. The study has found that the activated JNK pathway can significantly inhibit the differentiation of mesenchymal cells into adipocytes and promote their potential to differentiate into osteoblasts [99]. The JNK signaling pathway regulates methionine adenosyltransferase to promote the formation of mineralized calcium nodules in BMSCs and the expression of ALP, RUNX2, OCX, OCN, and DSPP genes [100]. Histone deacetylase inhibitor trichostatin A promotes the proliferation and differentiation of DPSCs through the JNK pathway. Furthermore, the activated JNK signaling pathway also plays a key role in calcium silicate-induced osteogenic differentiation of mesenchymal cells and DPSCs [101].

In addition to the mentioned signaling pathways, several classical pathways, including TGF-*β*, BMP-Smads, FGF, Rankl/OPG, and hypoxia-inducible factor-12, regulate DPSCs differentiation and osteogenesis. Notably, the nuclear factor-kappa B (NF-*κ*B) signaling pathway can promote or inhibit the regulation of DPSC differentiation depending on the specific stimulatory factors involved. For example, TNF-*α* [102] and estriol [103] have been shown to promote osteoblast differentiation by activating NF-*κ*B and upregulating the expression of ALP and BMP2. Conversely, IL-17 inhibits the proliferation and osteogenic differentiation of MSCs through NF-*κ*B [104]. The CaMKIV/CREB pathway can upregulate RUNX2 and downregulate PPAR*γ* expression by participating in Wnt5a, effectively promoting osteogenic differentiation [105] and inhibiting adipogenic differentiation [106]. Additionally, different scaffold materials have been found to activate the CaMKII pathway, inducing the proliferation and osteogenic differentiation of BMSCs [107–109]. However, whether CaMKII is involved in regulating the proliferation and differentiation of DPSCs is still unclear.

Overall, the regulation of osteogenic differentiation in DPSCs by signaling pathways is highly complex, exhibiting both synergistic and antagonistic effects. Thus, selecting an appropriate exogenous induction environment that directs the signaling pathway toward osteogenesis may prove more advantageous for DPSC-based BTE. However, at present, there remains a need for further research into the regulatory mechanism between upstream and downstream signaling pathways, the interrelationship among signaling pathways, and whether certain pathways known to act on MSCs can also be applied to DPSCs.

### 3.3. Scaffold Species

**Figure 4:**
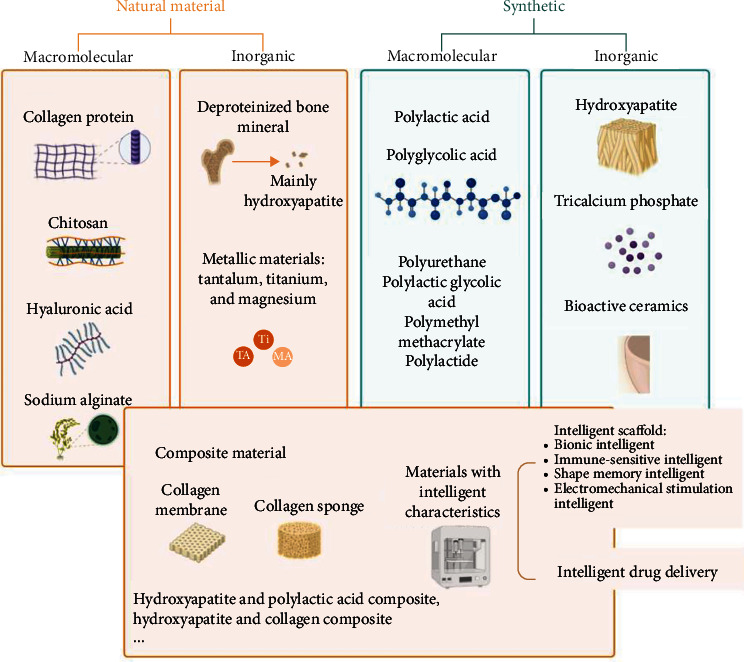
Bone tissue material scaffold. Degradable materials: polylactic acid, polyglycolic acid, copolymer polylactic glycolic acid, collagen sponge, collagen membrane, hydroxyapatite and polylactic acid composite, hydroxyapatite, and collagen composite. These materials will decompose in the human body and be removed by metabolites. Nondegradable materials: tantalum, titanium, magnesium, polymethyl methacrylate, polyurethane, polylactide, polyglycolide, polycaprolactone, hydroxyapatite, tricalcium phosphate, and bioceramics. These materials will not decompose in the body but will always exist.

To enhance the osteogenic differentiation of DPSCs, it is imperative to consider not only the growth factors and signaling pathways involved but also the appropriate scaffold species (Figure 4) of induction. Induction can be categorized into physical, chemical, and biological aspects based on the method employed. Physical induction methods involve creating a hypoxic environment [110, 111], pretreating DPSCs, or applying orthodontic load [112] to promote DPSCs osteogenic differentiation and self-renewal. In the chemical aspect, drug stimulation, such as the use of statins [113], aspirin [114], and estradiol, can promote osteoblast proliferation, differentiation, and inhibition of osteoclast formation.

The scaffold materials constitute an essential component of BTE, proving mechanical support, tissue shaping, and cytokines carrier. Collagen sponge or collagen membrane [115–117], as well as hydroxyapatite/tricalcium phosphate (HA/TCP) [15, 118–120] or HA/TCP ceramic [121, 122], is among the most commonly used scaffold types. Several studies have explored scaffolds composed of only HA [45, 83, 123], *β*-tricalcium phosphate (*β*-TCP) [124–127], PLGA membranes [128, 129], deproteinized bone mineral [130], etc. A 2018 systematic review tallied frequently used scaffold models [131]. Recently, biomaterials and structures with intelligent properties have been developed to better improve tissue regeneration and repair processes and improve tissue regeneration efficiency [132]. It mainly includes bionic intelligent scaffold [133] (bionic porous PLGA microspheres and peptide-coupled scaffold), immune-sensitive intelligent scaffold [134, 135] (amino-functionalized bioactive glass scaffold), shape memory intelligent scaffold [136] (shape memory porous nanocomposite scaffold composed of poly (*ε*-caprolactone) and HA nanoparticles), and electromechanical stimulation intelligent scaffold [137] (electrostatic spinning polyvinylidene difluoride-trifluoro ethylene fiber scaffold of zinc oxide nanoparticles). In addition, intelligent scaffolds can also be used for intelligent drug delivery [138].

In general, scaffolds are employed to promote the osteogenic differentiation of DPSCs. The biological effects of scaffolds on DPSCs are contingent on the specific type of bone defect and culture environment. Ideal scaffold tissue should exhibit strong biocompatibility, with a biodegradation rate matching the rate of new tissue regeneration. Consequently, selecting an appropriate scaffold is critical to achieving positive outcomes for DPSCs in BTE.

## 4. Application in BTE

The potential application of DPSCs and SHED for bone regeneration using different animal experimental models and biological scaffold materials is shown in Table 4. Yuan et al. [114] conducted a review of nearly 30 years of published studies to evaluate the in vivo BTE potential of DPSCs and SHED. The study considered the selection of animal models used in bone regeneration and repair research, which resulted in the following relative frequency order: mice (44%), rats (36%), rabbits (5%), pigs (4%), goats (2%), and dogs (2%).

### 4.1. Animal Experiment

Most scholars use rodent models to investigate the efficacy of DPSCs in promoting bone regeneration. Research on skull defects has shown that SHED can induce bone formation and differentiation into osteoblast-like cells in vivo [104]. Similarly, DPSCs can also differentiate into osteoblasts [102]. In ectopic osteogenesis models, SEHD can induce in vivo differentiation into bone when combined with suitable scaffolds [15]. Moreover, in the MRL/lpr (systemic lupus erythematosus) mice model, SHED can increase bone density and improve osteoporosis to promote bone regeneration [139, 140]. SHED has also shown potential as a new method for treating cleft lip and palate in rat maxillary alveolar bone defect models [115, 141]. When compared with BMSCs in an immunodeficient mouse skull defect model, SHED was found to be more conducive to the repair of alveolar clefts [26, 142].

The researchers have constructed a skull defect model [143, 144], a bone defect distraction osteogenesis model, mandibular bone defect model [145], and tibial bone defect model [59] in experimental rabbits, all of which demonstrated the effectiveness of DPSCs. In a pig mandibular defect model, the researchers implanted DPSCs to evaluate the new bone formation rate in the defect area, revealing that DPSCs can facilitate bone regeneration when added to the graft [109]. The application of DPSCs in a porcine periodontitis model, in combination with hepatocyte growth factor, exhibited the potential to promote periodontal bone regeneration and tissue repair [105]. The utilization of different scaffolds in the canine mandibular defect model [146, 147], sheep femoral head necrosis [148], and sheep femoral noncritical bone defect model [149] also confirmed the proliferation, osteogenic ability, and potential of DPSCs for repairing bone defects.

The animal model of bone regeneration needs to consider many factors, ignoring the economic conditions only for experimental purposes. The skull defect model is preferred for small rodents [150], and the adult sheep [149] is preferred for the long bone segmental defect. Because of its similar weight and bone remodeling rate and suitable size to adults, the results are more suitable for guiding clinical practice. The mandibular defect involving the oral cavity is better to choose the pig's mandible [151]. Of course, large animal models are the best preclinical simulation, while small animal models are more applied in terms of economy and time. At present, it is still necessary to further explore the realization of standardized models to eliminate or minimize uncertainties.

### 4.2. Clinical Application

In recent years, DPSCs have been utilized in clinical trials for BTE. These trials have primarily focused on the assessment of the efficacy of DPSCs in various anatomical regions of the human body. For instance, in one study, researchers transplanted DPSCs and collagen scaffolds into the bone defect area of patients with chronic periodontitis. After a follow-up period of 6 and 12 months, the experimental group demonstrated greater bone regeneration than the collagen sponge group [116]. Furthermore, DPSCs-IPs extracted from inflamed pulp tissue have also been shown to promote bone regeneration in the root bifurcation lesion area of patients with periodontitis [126]. Similarly, several scholars have reported that mixed transplantation of DPSCs and scaffold materials can promote the regeneration of alveolar bone [152, 153]. In another study, autologous DPSCs and HA-collagen sponges were implanted into the alveolar cleft area of patients with cleft palates to close the cleft [154]. The experimental group showed significantly better new bone mass and postoperative outcomes than the BMP2 group and the iliac bone transplantation group. Moreover, if low-power laser stimulation is applied concurrently, the osteogenic potential of DPSCs in cleft palate repair can be substantially improved [155].

However, in Carinci et al.'s study [156], no significant difference was observed after 6 months in the new bone density and the height of the apical septum of the extraction socket between the implanted group with DPSCs and collagen alone, which was contrary to previous studies. Despite the positive outcomes of most clinical trials, the clinical application of DPSCs still requires further high-quality trials in the future. As clinical problems in DPSCs application become more complex and require comprehensive consideration, it is critical to first design and plan the study. This includes careful consideration of application scenarios and standardized sampling in the planning phase. Additionally, determining optimal dosage and an effective matching method is essential. The number of cells used is determined by the dosage, while the degree of matching has a substantial impact on the patient's immune response and therapeutic outcomes. Proper use of appropriate scaffolds, cell sources, and growth factors is imperative in the osteogenic differentiation of DPSCs culture. Furthermore, exploring the related mechanisms of DPSC transplantation into the human body, the rejection of allogeneic DPSCs, and the long-term clinical effects postapplication are crucial for successful clinical application.

## 5. Conclusions

DPSCs possess multipotential differentiation ability, superior proliferation and self-renewal potential, low immunogenicity, and osteogenic ability in bone regeneration experiments, demonstrating their potential for further exploration in the field of BTE. Nevertheless, the mechanism of osteogenesis and related pathways associated with DPSCs remains elusive. Currently, it is crucial to further assure the safety of MSCs after transplantation, including genetic instability, tumorigenesis, and other related issues. Moreover, studies on DPSCs are mainly limited to animal and in vitro experiments, and high-quality clinical studies are still required to verify their advantages in future applications. Furthermore, the directed differentiation ability of DPSCs depends on the selection of appropriate scaffolds; it is essential to establish a comprehensive set of application standards for the processing of DPSCs and the selection of scaffolds after transplantation to maximize their efficacy and application safety.

## Figures and Tables

**Table 1:** The other sources of stem cells are from the oral cavity besides DPSC.

SCs	Sources
PDLSCs	Periodontal ligament
SCAPs	The apical papilla of an impacted tooth
GMSCs	Gingiva
ABMSCs	Alveolar bone
TGPCs	Tooth germ
DFSCs	Dental follicles

PDLSCs, periodontal ligament stem cells; SCAPs, stem cells from the apical papilla; GMSCs, gingiva-derived mesenchymal stem cells; ABMSCs, alveolar bone-derived mesenchymal stem cells; TGPCs, tooth germ progenitor cells; DFSCs, dental follicle progenitor cells.

**Table 2:** Isolated cell marker expression.

Markers	DPSC	SHED
Positive markers	CD271, CD166, CD146, CD106, CD105, CD90, CD73, CD59, CD49, CD44, CD29, CD13, CD10, CD9, Stro-1, and nestin	CD166, CD146, CD105, CD90, CD73, CD56, CD44, CD29, CD13, Stro-1, and nestin

Negative markers	CD133, CD117, CD7, CD45, CD34, CD33, CD31, CD24, CD19, CD14, CD11b, CD8, and CD3	CD45, CD43, CD34, CD19, CD14, and CD11b

**Table 3:** The main role of the growth factors.

Growth factor	Main mechanism
TGF-*β*	Osteogenic and chondrogenic differentiation
BMPs	Chondrogenic, osteogenic, and osteoinductive
bFGF	Angiogenesis, proliferation, and osteogenic differentiation
NGF	Promoting bone metabolism and osteogenic differentiation
PDGF	Osteogenic and endothelial differentiation with gene expression
IGF	Anabolic and catabolic effects on osteogenesis
VEGF	Osteoinductive, chemotactic, and angiogenesis
FGF	Angiogenesis, proliferation, and osteogenic differentiation

TGF-*β*, transforming growth factor-*β*; BMPs, bone morphogenetic proteins; BFGF, basic fibroblast growth factor; NGF, nerve growth factor; PDGF, platelet-derived growth factor; IGF, insulin-like growth factor; VEGF, vascular endothelial growth factor; FGF, fibroblast growth factor.

**Table 4:** Application of DPSCs and SHED for BTE in different animal models and scaffolds.

Author	Animal model	Stem cells	Scaffold	Mode of transplantation
Miura et al. [15]	Immunocompromised mice	SHEDs	HA/TCP	Subcutaneous implantation
Li et al. [126]	MRL/Ipr mice	SHEDs	HA/TCP	Intravenous administration
Beztsinna et al. [107]	Immunocompromised mice	DPSCs	3D Bioglass®	Intraperitoneal implantation
Keller et al. [105]	Rats	DPSCs	HA/TCP	Cranial defect
Jang et al. [102]	Rats	SHEDs	Collagen matrix	Maxillary alveolar bone defect
Ghavimi et al. [128]	Rats	DPSCs	Woven bone (WB)	Mandibular bone defect
Du et al. [129]	Immunodeficient mice	SHEDs/hDPSCs/hBMSCs	Polylactic-coglycolic acid barrier membrane	Calvaria defect
Hu et al. [134]	Dogs	SHEDs	Cell-free collagen scaffold	Mandibular bone defect
Chen et al. [135]	Sheep	DPSCs	None	Femoral defects
Chan et al. [136]	Sheep	DPSCs	Bonelike®	Femoral defects
Zheng et al. [108]	Pigs	DPSCs	HA/TCP	Periodontal bone defect
Wu et al. [111]	Pigs	DPSCs	CSD, *α*-CSH/ACP, and *β*-TCP scaffold	Mandibular bone defect

## Data Availability

The data used to support the findings of this study are available from the corresponding author upon request.
